# Discovery of dual S-RBD/NRP1-targeting peptides: structure-based virtual screening, synthesis, biological evaluation, and molecular dynamics simulation studies

**DOI:** 10.1080/14756366.2023.2212327

**Published:** 2023-05-17

**Authors:** Chunfang Hu, Ting Guo, Yunting Zou, Junyi Gao, Yi Gao, Miaomiao Niu, Yang Xia, Xiaozhou Shen, Jindong Li

**Affiliations:** a Institute of Clinical Medicine, The Affiliated Taizhou People’s Hospital of Nanjing Medical University, Taizhou, China; b Institute of Clinical Medicine, Taizhou People’s Hospital Affiliated to Nanjing University of Traditional Chinese Medicine, Taizhou, China; c Department of Pharmaceutical Analysis, China Pharmaceutical University, Nanjing, China

**Keywords:** SARS-CoV-2, receptor-binding domain, neuropilin-1, dual inhibitors, virtual screening

## Abstract

Both receptor-binding domain in spike protein (S-RBD) of severe acute respiratory syndrome coronavirus 2 (SARS-CoV-2) and human neuropilin-1 (NRP1) are important in the virus entry, and their concomitant inhibition may become a potential strategy against the SARS-CoV-2 infection. Herein, five novel dual S-RBD/NRP1-targeting peptides with nanomolar binding affinities were identified by structure-based virtual screening. Particularly, RN-4 was found to be the most promising peptide targeting S-RBD (*K*_d_ = 7.4 ± 0.5 nM) and NRP1-BD (the b1 domain of NRP1) (*K*_d_ = 16.1 ± 1.1 nM) proteins. Further evidence in the pseudovirus infection assay showed that RN-4 can significantly inhibit the SARS-CoV-2 pseudovirus entry into 293 T cells (EC_50_ = 0.39 ± 0.09 μM) without detectable side effects. These results suggest that RN-4, a novel dual S-RBD/NRP1-targeting agent, holds potential as an effective therapeutic to combat the SARS-CoV-2 infection.

## Introduction

On 30 January 2023, world health organisation (WHO) declared that coronavirus disease 19 (COVID-19) caused by severe acute respiratory syndrome coronavirus 2 (SARS-CoV-2) is still a public health emergency of international concern (PHEIC). As of 28 February 2023, about 758 million confirmed cases and 6.8 million deaths have been reported (https://covid19.who.int/). For the treatment of virus infection, single-target drugs are becoming less effective due to drug resistance and the multiple regulatory proteins in the process of infection^1^. Clinically, the combination of two or more drugs acting on different pathways has been successfully used in virological arena^2^^,^^3^. However, such combination therapy may be hindered by possible drug − drug interactions, poor patient compliance and cumulative toxicities^4–6^. Hence, the dual-targeting strategy has gained great attention in antiviral drug discovery. In this strategy, a dual-targeting molecule is designed to interact with multiple proteins, thereby improving efficacy and reducing side effects^7^^,^^8^. In this work, we took two important regulatory proteins in the process of SARS-CoV-2 entry into host cells as key targets, and identify the dual-targeting inhibitors through a computer-aided drug design strategy to prevent the SARS-CoV-2 infection.

SARS-CoV-2 is a single-stranded ribonucleic acid (RNA) virus, consisting of spike (S), membrane (M), envelope (E), and nucleocapsid (N) proteins^9–12^. Studies have shown that receptor-binding domain (RBD) in the S1 subunit of S protein is a key regulatory protein that mediates the entry of the virus into the host cell because it can interact with angiotensin-converting enzyme 2 (ACE2) on the host cell membrane, thereby effectively triggering the membrane fusion between the virus and the host cell^13–15^. Recently, many agents were found to be able to bind with S-RBD and have anti-SARS-CoV-2 activity such as demethylzeylasteral (T-96), glycyrrhizic acid, and cepharanthine^16–18^. Therefore, the S-RBD-targeting inhibitors which block the interaction between S-RBD and ACE2 may be an efficient countermeasure to combat COVID-19^19^^,^^20^.

**Figure 1.**
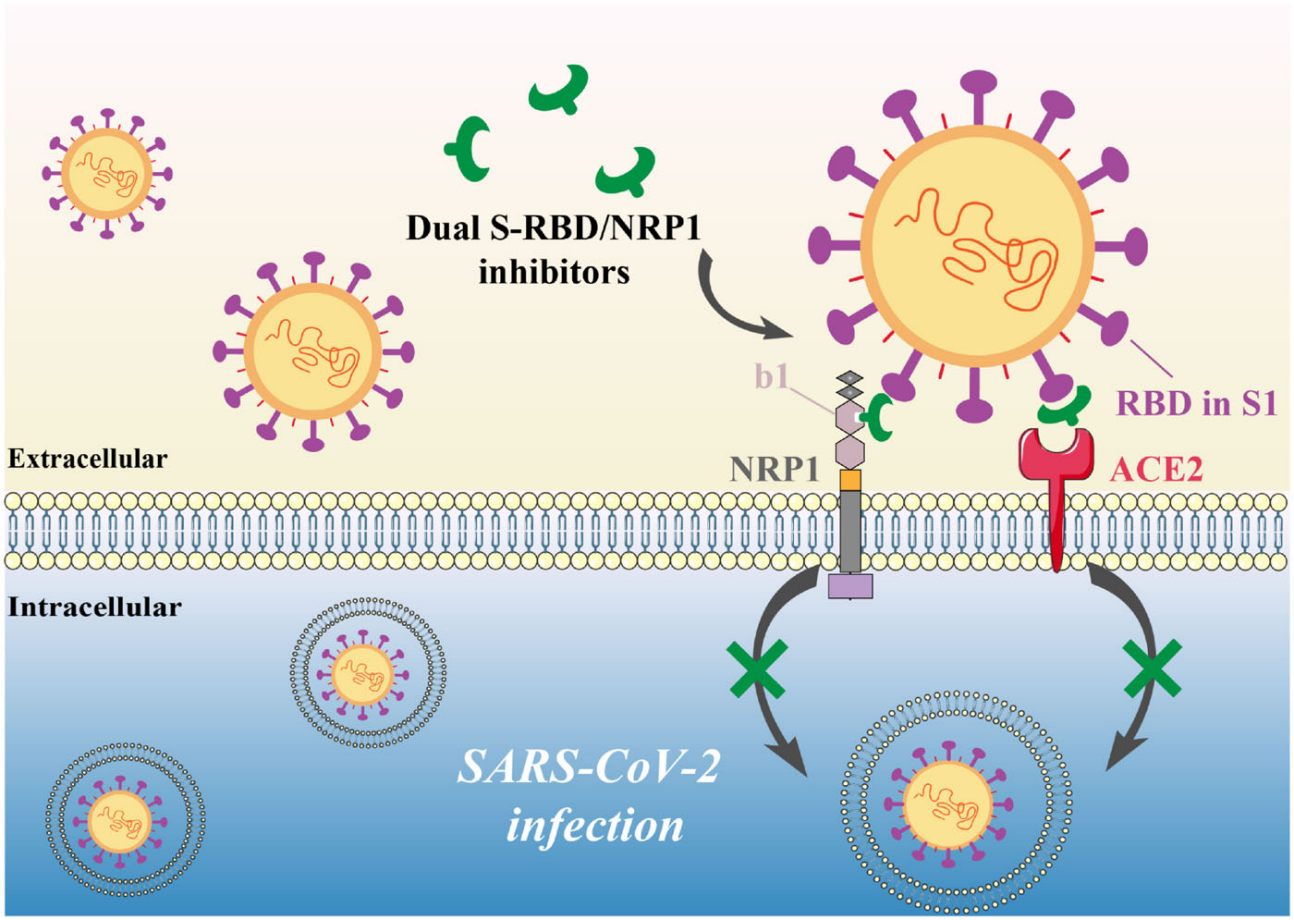
Anti-SARS-CoV-2 mechanism of S-RBD/NRP1 dual-targeting inhibitors.

Recent studies have shown that neuropilin-1 (NRP1) is another key regulatory factor for SARS-CoV-2 to enter the host cell, which helps to enhance the infectivity of SARS-CoV-2^21^^,^^22^. NRP1 is a multifunctional transmembrane protein, mediating various physiological processes such as angiogenesis and the development of neurons^23–25^. It includes a transmembrane domain, an extracellular domain (composed of a1/a2, b1/b2, and c domains), and a small cytoplasmic domain (Cyt)^26^. Among them, the b1 domain is often used as the target to design NRP1 inhibitors such as EG00229 and EG01377^26^^,^^27^. Daly et al. established that NRP1 can increase viral infectivity because the b1 domain can directly bind to the amino acid sequence (^682^RRAR^685^) in the cleaved S1 protein^28^. Cantuti-Castelvetri et al. suggested that NRP1 may form a co-receptor with ACE2 to enhance the virus-host cell attachment in respiratory and olfactory epithelial cells, so as to facilitate viral entry^29^. Moreover, NRP1 is found to be expressed in olfactory tubercles and para-olfactory gyri, among which ACE2 is expressed at a low level, suggesting that NRP1 may be another regulatory factor for SARS‑CoV‑2 infection involved in the neurologic manifestations of COVID-19^30^. Based on these findings, simultaneously inhibiting S-RBD and NRP1 may result in a synergistic effect on the inhibition of virus infection, and dual S-RBD/NRP1-targeting inhibitors may become a potential therapeutic approach to preventing SARS-CoV-2 infection. Therefore, in this work, we aimed to discover dual S-RBD/NRP1-targeting anti-SARS-CoV-2 inhibitors as shown in Figure 1. Notably, no dual S-RBD/NRP1-targeting inhibitors are currently available.

Although the poor stability in the circulating blood and immunogenicity have limited the effective application of peptides, they have larger surfaces and more functional groups compared to small molecules, which enable them to fully disrupt the protein-protein interactions^31^^,^^32^. Besides, peptides are often with low toxicity and high efficiency^33^. In this work, we have successfully found five promising dual-targeting peptide inhibitors of S-RBD and NRP1 using pharmacophore-based docking and structure-based docking. Then, the five selected peptides were synthesised and bio-evaluated. Among them, RN-4 simultaneously exhibited the strongest binding affinities to S-RBD and NRP1 *in vitro* and further showed obvious inhibitory effects against SARS-CoV-2 pseudovirus into cells. These data suggest that RN-4 is a promising dual S-RBD/NRP1-targeting peptide to treat the infection of SARS-CoV-2.

## Materials and methods

### Structure-based pharmacophore modelling

The Molecular Operating Environment (MOE, Chemical Computing Group Inc, Montreal, Quebec, Canada) program was employed in the following *in silico* drug screening operations. The crystal structure of SARS-CoV-2 S-RBD bound to ACE2 downloaded from Protein Data Bank (PDB) (www.rcsb.org, PDB ID: 6M0J) was imported into MOE and the Prepare Protein tool was used to pre-process the structure by adding hydrogen atoms. Then intermolecular interactions between S-RBD and ACE2 are visualised and analysed by the Ligand Interactions module of MOE. Based on the analysis, the Pharmacophore Editor was applied to create the most representative features, which indicated the key interaction points for the ligand binding to S-RBD. Furthermore, the Güner–Henry (GH) scoring method was employed to validate the selectivity of S-RBD pharmacophore model. The virtual screening of a testing set database was carried out using the pharmacophore search protocol of MOE. The hit lists were analysed based on the following formula:

$$GH=(\frac{Ha(3A+Ht)}{4HtA})(1-\frac{\left(Ht-Ha\right)}{\left(D-A\right)})$$

Statistical parameters including total molecules in the database (*D*), Total number of actives in database (*A*), total hits (*Ht*), active hits (*Ha*), Enrichment factor (*E*), and goodness of hit score (*GH*) were calculated. The *GH* score ranges from 0 (indicating a null model) to 1 (indicating an ideal model).

### Virtual screening

The crystal structure of the b1 domain of NRP1 (NRP1-BD) was obtained from PDB (PDB ID: 7JJC) and preprocessed via Prepare Protein tool. The virtual library containing 24 000 peptides was self-constructed using the QuaSAR-CombiGen module of MOE. This module created a random peptide by linking nonapeptide fragments, heptapeptide fragments, dodecapeptide fragments, and tetrapeptide fragments; see the reported article for more details^34^. The converting of each peptide structure from 2D to 3D was achieved by the use of the Energy Minimisation algorithm. Based on the generated pharmacophore model of S-RBD, a pharmacophore-based docking simulation was conducted to identify S-RBD-targeting peptides by the Docking module of MOE. Key residues on the binding surface of S-RBD were selected as a binding site. The pharmacophore-base docking algorithm served to dock peptides into the S-RBD binding site. The binding free energy was calculated by the dG docking scoring. The selected peptides with docking scores lower than −13.5 kcal/mol subsequently docked into the NRP1-BD active site with the triangle matcher algorithm. Finally, according to the dG docking scores, the top five peptides were selected for *in vitro* biological testing.

### Synthesis of the peptides

The method of solid-phase peptide synthesis was described previously^35^. The manufactured peptides were then identified utilising high-performance liquid chromatography (HPLC) and mass spectrometry (MS). In this study, the purity of these peptides is more than 98% by HPLC analysis. The HPLC chromatogram and MS spectrum were provided in the Supporting information.

### Microscale thermophoresis (MST) analysis

Recombinant SARS-CoV-2 S-RBD and NRP1-BD proteins were purchased from Abcam (Cambridge, MA, USA). The Monolith NT.115 instrument (Nanotemper Technologies GmbH) was used for this experiment, and the buffer (pH 7.0) contained Tris (50 mM) and NaCl (230 mM). The Lys labelling kit was applied to label commercial S-RBD and NRP1 for detection, with a final concentration of 50 nM of each labelled peptide. The peptides were diluted 1:1 and titrated starting at 12.5 μM (according to the solubility of each peptide). Following 5 min of centrifugation at 15 000 rpm, the binding reaction product was obtained and added to a standard glass capillary. The automatically allocated power of 20% LED and 50% MST was used in all MST measurements.

### Cell culture

Human embryonic kidney 293 T cells (293 T), human pulmonary alveolar epithelial cells (A549), and human foetal small intestinal epithelial cells (FHs 74 Int) were purchased from American Type Culture Collection (ATCC) (Manassas, VA, USA), and human cardiomyocyte cells (AC16) were purchased from Millipore (NY, USA). The Dulbecco’s modified Eagle’s medium (DMEM) was used to culture these cell lines under a humidified atmosphere (95%), 5% CO_2_ at 37 °C. This medium contained 1% penicillin–streptomycin and 10% foetal bovine serum (FBS).

### Pseudovirus affinity and infection

To test the infection-blocking effect of peptides against SARS-CoV-2, pseudovirus affinity and infection experiments were conducted. The SARS-CoV-2 pseudovirus was derived from pseudotyped HIV-1 virus expressing S protein of SARS-CoV-2. To be detectable, the pseudovirus also contained a firefly luciferase gene. 293 T cells at a 2 × 10^4^ density per well were seeded in 96-well plates and incubated overnight at 37 °C. The diluted ligands were preincubated with the pseudovirus for 1 h at 37 °C before being transferred to 293 T cells. Following 48 h of incubation, the cells were washed with 100 μl of PBS. 50 μl of lysis buffer was used to lyse cells. The luciferase detection reagent was included in this lysis buffer, and signals would be detected by using the luciferase detecting kit (according to instructions of manufacturer, Promega).

### Cytotoxicity assay

The cell seeding concentration was 5 × 10^4^ cells/mL, cultivating in 96-well plates. Following the incubation overnight at 37 °C in a 5% (v/v) CO_2_ atm, cells were treated for 48 h at various concentrations of RN-4 (0, 5, 10, 20, 40, and 80 μM). Then, the cells were incubated for 4 h after addition of MTT stock solution (0.5 mg/mL). During 2 h at 37 °C, cells were lysed in lysis buffer. Using a microplate reader, the absorbance was evaluated at 570 nm.

### Molecular dynamics (MD) simulations

The S-RBD-RN-4 and NRP1-BD-RN-4 complexes were subjected to simulating by GROMACS 2019.4 in the AMBER99SB-ILDN force field with periodic boundary conditions. Firstly, solving the complex in a cube box with 1.0 nm distance away from the complex by single point charge (SPC) water molecules, and the system was neutralised by replacing water molecules with Na^+^ and Cl^–^. Then, energy minimisation of the system was conducted for 1500 steps using the steepest descent algorithm. NVT simulation was further conducted for 100 ps with a V-rescale thermostat to maintain the temperature of the system at 300 K. Later, 100 ps NPT simulation was performed using Parinello–Rahman barostat to maintain the pressure of the system 1 bar. Finally, the system was subjected to 100 ns MD simulations. Trajectory data were saved at time intervals of 100 ps.

## Results

### Structure-based pharmacophore modelling

**Figure 2.**
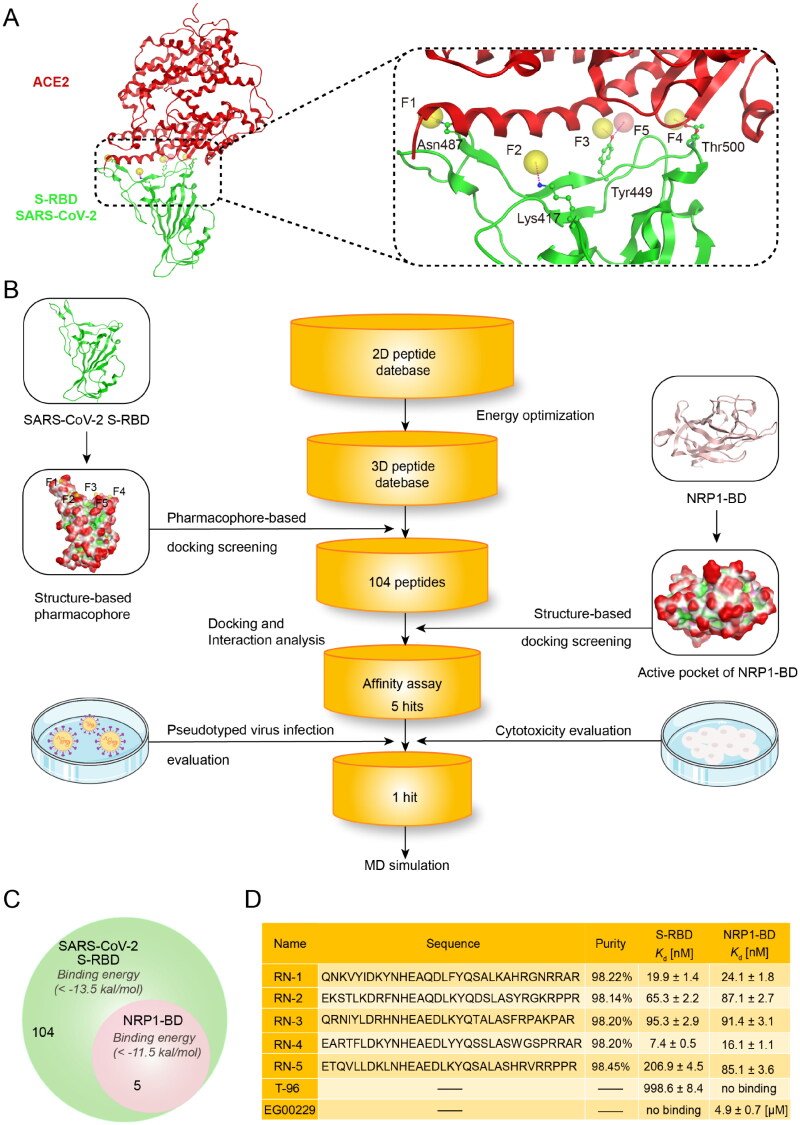
(A) Pharmacophore model of S-RBD. The residues in active site of S-RBD were presented as sticks with atoms coloured carbon-green, oxygen-red, and nitrogen-blue. (B) Workflow of the multistep computer-aided screening of dual S-RBD/NRP1-targeting peptides. (C) Identification of five peptides with lower S-RBD-binding energies and NRP1-BD-binding energies. (D) Sequences and properties of RNs 1–5. Binding affinity of RNs 1–5 to S-RBD and NRP1-BD. Results shown represented the mean ± SD (*n* = 3). The N-terminus of RNs 1 − 5 was acetylated.

Pharmacophore model depicts the three-dimensional (3D) properties chemically and structurally that a ligand must possess to ensure the optimum ligand-receptor binding^36^. Nowadays, the pharmacophore-based approach has been successfully employed to discover novel and potent peptides to obstruct protein − protein interactions with large and shallow protein-binding interfaces^37^. Since S-RBD binding surface is wide and lacks a deep pocket, a pharmacophore model was created in this study to improve the identification of potential S-RBD-inhibitory peptides. Previous research has demonstrated that the conformation of S-RBD converts between the “down” and “up” states and S-RBD can interact with ACE2, forming a well-defined pocket on their interface only in “up” conformation^38^. Thus, the structure of S-RBD co-crystallised with ACE2 (PDB ID: 6M0J) was applied as the templet for pharmacophore generation because the “up” conformation of S-RBD is possessed. The S-RBD-ACE2 interactions were analysed by Ligand Interactions module of MOE. We found that interactions between S-RBD and ACE2 were mediated primarily by polar interactions with some critical amino-acid residues of S-RBD: Asn487, Lys417, Tyr449, and Thr500. Therefore, an S-RBD pharmacophore model containing four hydrogen-bond acceptor features (F1 − F4: Acc) and one hydrogen-bond donor feature (F5: Don) was created by the Pharmacophore Editor module of MOE (Figure 2(A)). All of these features characterised hydrogen-bond interactions between ligand and S-RBD: (i) the F1 feature corresponded to Asn487; (ii) the F2 feature corresponded to Lys417; (iii) both F3 and F5 features corresponded to Tyr449; (iv) the F4 corresponded to Thr500. Notably, the putative peptides should present hydrogen-bond acceptor and donor features simultaneously to interact with Tyr449. Moreover, given that the active pocket of S-RBD is wide and large, the F1 and F4 features were located at two ends of the binding surface to identify peptides that can block the whole S-RBD binding surface and fully hinder interactions between S-RBD and ACE2. In order to verify the ability of S-RBD pharmacophore model to identify active inhibitors from database, the model was further evaluated using the Güner–Henry (GH) score method^39^. In this validation process, a test set database consisted of 494 inactive peptides and 6 active derivative peptides derived from ACE2 protein was screened by the pharmacophore model^40^. The following statistical parameters were computed to analyse the screening results, including total hits (*Ht*), enrichment factor (*E*), and goodness of hit score (*GH*). The results were shown in Table S1 (Supporting information). Generally, the GH score between 0.7 and 1 means that the validated model is very good. It was observed that the GH score of S-RBD pharmacophore model is 0.81, indicating the good capacity of S-RBD pharmacophore model to distinguish active inhibitors from inactive ones.

### Virtual screening

The virtual screening method used in this study is shown in Figure 2(B). To screen novel S-RBD/NRP1 dual-targeting inhibitors from the independently constructed peptide database, we combined pharmacophore-based molecular docking with structure-based molecular docking. Firstly, we constructed a two-dimensional (2D) peptide database containing 24 000 peptides. In order to perform the virtual screening of dual-targeting S-RBD/NRP1 inhibitors, we converted the 2D database into 3D structures through the energy optimisation protocol of MOE. Then, to filter S-RBD-target peptides, the constructed model of S-RBD was applied as a 3D query and key residues on the binding surface of S-RBD were selected as the binding site to screen the database by using the pharmacophore-based molecular docking screening. The docking scoring was computed for each peptide to predict the binding affinity between the peptide and S-RBD (a lower score value suggesting a better binding affinity). The reliability of the docking protocol was verified before the docking screening. Before the docking screening, we used the wild-type peptide (^21^IEEQAKTFLDKFNHEAEDLFYQSSLASWNYNTNIT^55^) derived from the ACE2 protein from the RBD-ACE2 complex (PDB ID: 6M0J) as a template to validate the S-RBD docking protocol. As displayed in Figure S1 (Supporting information), the docking conformation (cyan) of the wild-type peptide could be well matched with the original conformation (red) in the active site of SARS-CoV-2 S-RBD, suggesting that the S-RBD docking protocol is reliable. Therefore, this docking protocol could be used in the S-RBD pharmacophore-based molecular docking screening. In this S-RBD docking process, the previous reported derivative peptide 5 derived from ACE2 protein and the compound T-96 were used as positive controls and their docking scores were −12.83 and −13.14 kcal/mol, respectively (Table S2)^16^^,^^40^. Thus, we chose a lower energy value of −13.5 kcal/mol as a cut-off value for the prediction of the binding affinity between peptides and S-RBD, and 104 peptides were obtained from the database. All of the 104 peptides could be mapped onto all the pharmacophore features of S-RBD and have the docking binding free energy < −13.5 kcal/mol (Figure 2(C)). Subsequently, in order to screen the dual S-RBD/NRP1-targeting peptides, the 104 selected S-RBD-targeting peptides were docked into the active site of NRP1-BD (PDB ID: 7JJC). Before the NRP1-BD docking screening, the co-crystallized ligand CendR peptide from the NRP1-BD crystal structure was used as a template to validate the docking protocol of NRP1-BD. As shown in Figure S2 (Supporting information), the docking conformation (cyan) of the co-crystallized ligand CendR peptide could be well mapped onto the original conformation (red) in the NRP1 active site, indicating that the NRP1 docking protocol is reliable. Based on the NRP1-BD docking results, we selected the top five candidate peptides, called as RNs 1–5, with NRP1-BD binding energy < −11.5 kcal/mol for further study (Figure 2(C) and Table S2, Supporting information).

### Identification of RNs 1–5 targeting S-RBD and NRP1-BD

We first conducted MST assay to determine the binding ability of RNs 1 − 5 to S-RBD and NRP1-BD proteins. The S-RBD targeting inhibitor T-96 and the NRP1 targeting inhibitor EG00229 were selected as positive controls^16^^,^^26^. The results in Figure 2(D) displayed that RNs 1–5 possessed excellent binding affinities to the S-RBD and NRP1-BD proteins, ranging from 7.4 to 206.9 nM and 16.1 to 91.4 nM, respectively (Figure 2(D)). Among them, RN-4 was the most potent peptide targeting S-RBD protein (*K*_d_ = 7.4 ± 0.5 nM) and NRP1-BD (*K*_d_ = 16.1 ± 1.1 nM). Moreover, the results displayed that T-96 showed a strong binding ability to S-RBD (*K*_d_ = 998.6 ± 8.4 nM) without binding to NRP1-BD, while EG00229 exhibited excellent binding affinity to NRP1-BD (*K*_d_ = 4.9 ± 0.7 μM) without binding to S-RBD (Figure 2(D)). Compared with T-96 and EG00229, the binding of RN-4 to the S-RBD and NRP1-BD proteins was increased remarkably by about 135- and 304-fold, respectively.

**Figure 3.**
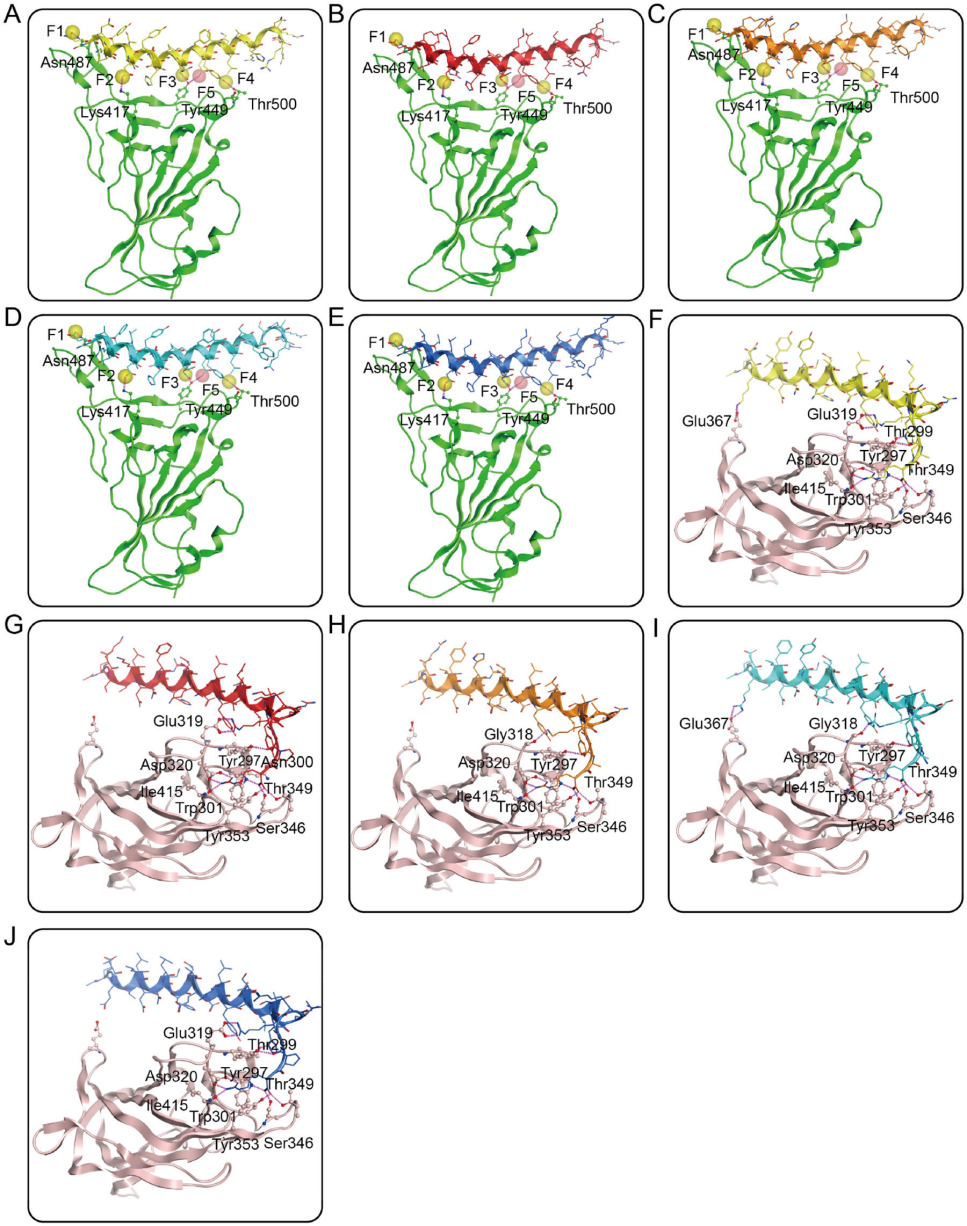
(A–E) Probable binding models of RNs 1–5 with S-RBD. Residues in S-RBD were presented as sticks with carbon atoms colour-coded by green, nitrogen atoms colour-coded by blue, and oxygen atoms colour-coded by red. (F-J) Probable binding models of RNs 1–5 with NRP1-BD. Residues in NRP1-BD were presented as sticks with carbon atoms colour-coded by light pink, nitrogen atoms colour-coded by blue, and oxygen atoms colour-coded by red. The RNs 1–5 were presented as sticks with nitrogen atoms colour-coded by blue, oxygen atoms colour-coded by red, and carbon atoms colour-coded by yellow, red, orange, cyan, and dark blue, respectively. The hydrogen-bond interactions were presented by purple dotted lines.

Next, the interactions of RNs 1–5 with active sites of S-RBD and NRP1-BD were analysed by a molecular docking study to explore the probable binding modes. Figure 3(A–E) showed the interactions between RNs 1–5 and S-RBD. The α-helix region with a similar type of orientation in each peptide played a paramount role in interacting with S-RBD by forming five hydrogen bonds with critical residues Asn487, Lys417, Tyr449, and Thr500, which were in great agreement with the established S-RBD pharmacophore features F1-F5. All of these S-RBD-peptides interaction analysis indicate that RNs 1–5 can match well with the constructed pharmacophore model and have good binding affinities to S-RBD. Figure 3(F–J) displays the binding models of RNs 1–5 with NRP1-BD. The C terminal motif R/K/XXR/K of each peptide was the main region binding to NRP1, which was the same as expected. Moreover, RNs 1–5 could create hydrogen bonds with residues Gly318/Glu319, Tyr297, Trp301, Asp320, Ser346, Thr349, Tyr353, and Ile415 of the NRP1-BD, most of which have been reported as key residues for interactions between NRP1-BD and the ^682^RRAR^685^ motif in SARS-CoV-2^28^. Collectively, these docking results suggest that RNs 1–5 can simultaneously have interactions with key residues of both S-RBD and NRP1-BD.

### Inhibition of pseudotyped SARS-CoV-2 infection

**Figure 4.**
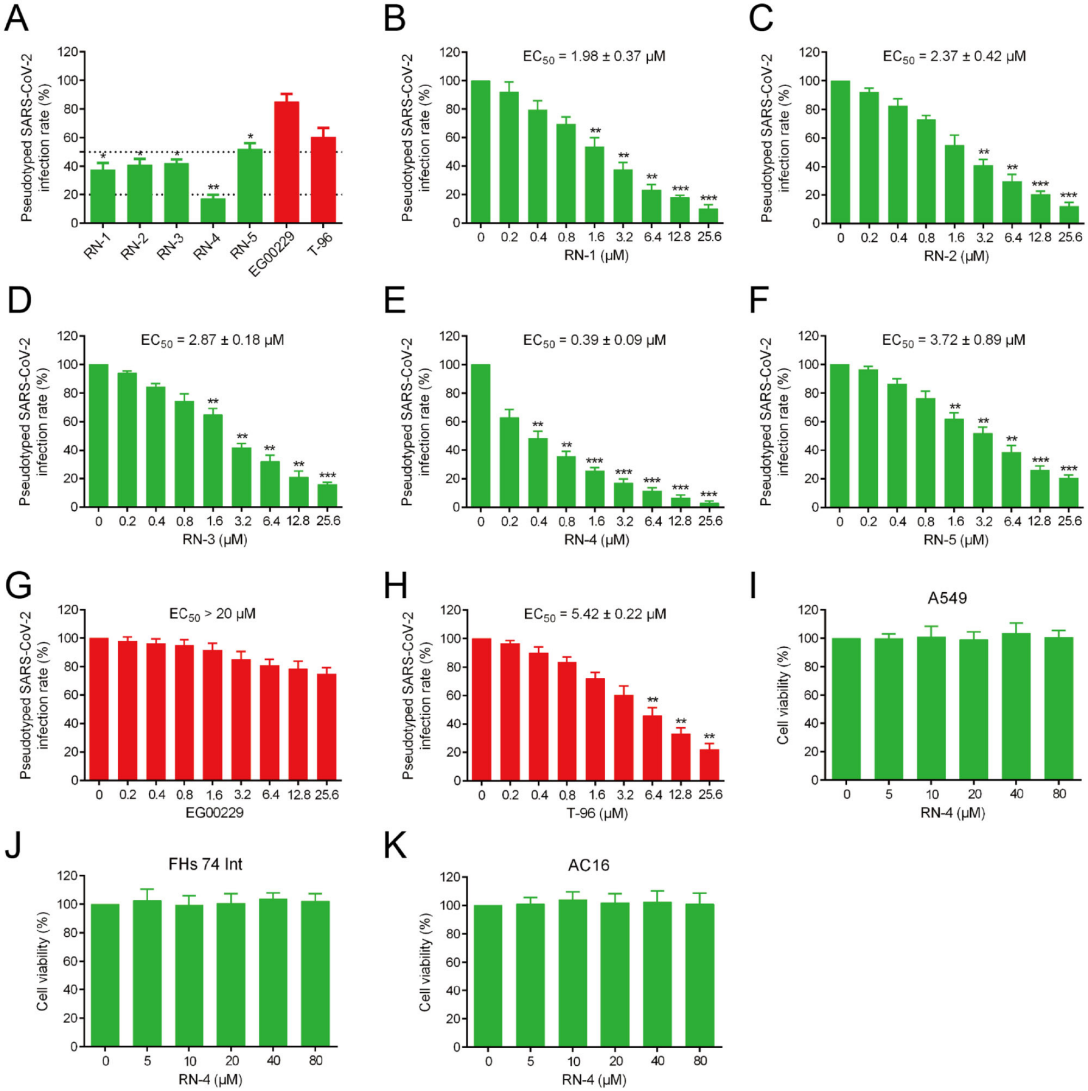
Infection assay of the screened peptides RNs 1–5 against pseudotyped SARS-CoV-2. (A) Infection rate of the pseudotyped SARS-CoV-2 in the presence of RNs 1–5, EG00229, and T-96 at a concentration of 3.2 μM. *p < 0.05, **p < 0.01 versus EG00229. (B-H) Dose-dependent (0–25.6 μM) assay of RNs 1–5, EG00229, and T-96. *p < 0.05, **p < 0.01, ***p < 0.001 versus control. (I-K) Effects of RN-4 on cell viability. A549, FHs 74 Int, and AC16 were incubated with RN-4 (0, 5, 10, 20, 40, and 80 μM, respectively) for 48 h. Data are presented as mean ± SD (*n* = 3).

At present, SARS-CoV-2 pseudovirus has been widely used for screening active compounds in anti-infection experiments because of its high security, strong stability, and wide host tropism^41^^,^^42^. In this study, a SARS-CoV-2 pseudovirus infection assay was conducted to detect the antiviral potencies of RNs 1–5. Firstly, the cytotoxicity of RNs 1–5 was evaluated by a MTT assay. The results showed that RNs 1–5 had no significant toxicity to 293 T cells at 30 µM concentration (Figure S3, Supporting information). Later, this study will carry out the experiment of inhibition of pseudotyped SARS-CoV-2 infection by RNs 1–5 at non-toxic concentration. The results in Figure 4(A) displayed that the inhibition rate of RN-5 on viral infection was slightly less than 50%, while the inhibitory effects of RNs 1–4 on pseudotyped SARS-CoV-2 virus entry was more than 50% at 3.2 µM concentration. The inhibition rate of RN-4 on SARS-CoV-2 infection was ≥80%, showing the strongest inhibition activity. Moreover, the inhibition rate of the NRP1 inhibitor EG00229 at the concentration of 3.2 µM is only 15%, while the inhibition rate of S-RBD inhibitor T-96 is about 40%. Furthermore, the results showed that the corresponding EC_50_ values of RNs 1–5 and T-96 were 1.98, 2.37, 2.87, 0.39, 3.72 and 5.42 µM, respectively. EC_50_ value of EG00229 is more than 20 µM (Figure 4(B–H)). This result shows that compared with the single-target inhibitors T-96 and EG00229, the dual-target inhibitors can better inhibit the pseudotyped SARS-CoV-2 infection. It is noteworthy that the inhibition of RN-4 on SARS-CoV-2 pseudovirus infection is about 14 times higher than that of T-96. In the next experiment, we not only further investigate the biological safety of RN-4 through MTT assay but also investigate its dual-targeting binding mechanism through molecular dynamics simulation studies.

### Safety profiles of RN-4

According to recent studies, clinical manifestations of COVID-19 mainly contain respiratory symptoms, while gastrointestinal symptoms and severe cardiovascular damage may occur. In the relevant organs (lungs, intestines, and heart), SARS-CoV-2 virus was detected^43–45^. Hence, a MTT assay was performed to further explore the safety profiles of RN-4. The cytotoxicity of RN-4 to A549, FHs 74 Int, and AC16, which corresponded to the above symptoms of COVID-19, was detected. After being treated with RN-4 for 48 h, the viability of cells was hardly affected in a dose-dependent manner (Figure 4(I–K)). Importantly, RN-4 did not show any observable cytotoxicity even at the high concentration of 80 μM, demonstrating the excellent safety of RN-4.

### MD Simulations

**Figure 5.**
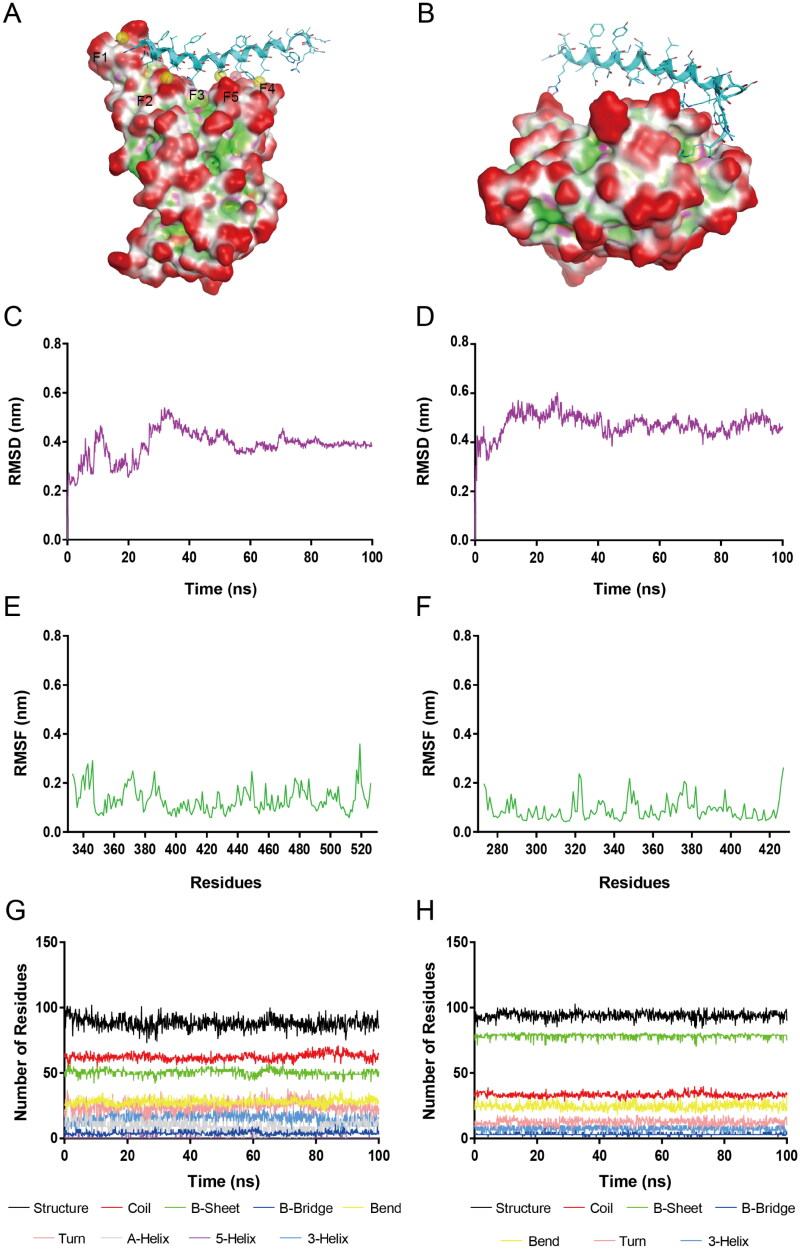
(A) The S-RBD-RN-4 complex for MD simulations. (B) The NRP1-BD-RN-4 complex for MD simulations. (C) RMSD of all atoms in the S-RBD-RN-4 complex. (D) RMSD of all atoms in the NRP1-BD-RN-4 complex. (E) RMSF of S-RBD backbone (Cα) atoms in the S-RBD-RN-4 complex. (F) RMSF of NRP1-BD backbone (Cα) atoms in the NRP1-BD-RN-4 complex. (G) Secondary structure of S-RBD in the S-RBD-RN-4 complex. (H) Secondary structure of NRP1-BD in the NRP1-BD-RN-4 complex.

Based on the results of *in vitro* biological testing, RN-4 was considered as the most potential S-RBD/NRP1 dual inhibitor. The S-RBD-RN-4 and NRP1-BD-RN-4 complexes (Figure 5(A, B)) were further accessed by 100 ns MD simulations with GROMACS 2019.4 to evaluate time-dependent binding modes, structural stability, and conformational fluctuations. In order to investigate the conformational stability of S-RBD-RN-4 and NRP1-BD-RN-4 systems and the flexibility of receptor proteins, RMSD, as well as root-mean-square fluctuation (RMSF), were calculated. RMSD represents the stability of a system during the whole process of MD simulation. The gmx_rmsd module of GROMACS was applied to calculate the RMSD value. The RMSD plots (nm) of S-RBD-RN-4 and NRP1-BD-RN-4 complexes for the atoms in 100 ns MD simulation are displayed in Figure 5(C and D), respectively. The RMSD of S-RBD-RN-4 had risen suddenly initially with an increase up to 30 ns and finally attained stability at about 0.4 nm after 70 ns. The RMSD of NRP1-BD-RN-4 had a burst rise followed by constant growth up to 18 ns and eventually stabilised between 0.4 nm and 0.5 nm, while a slight decrease in value was observed. These results indicate that RN-4 can stably bind to both S-RBD and NRP1-BD in the whole simulation process. Then RMSF of backbone (Cα) atoms was analysed to observe residues fluctuations and flexibility of S-RBD and NRP1-BD during 100 ns MD. The gmx_rms module was used to compute the RMSF value (the higher value indicates more flexibility). As shown in Figure 5(E, F), residues of S-RBD and NRP1-BD were not too flexible in the whole 100 ns simulation with fluctuation intensity <0.38 nm and 0.3 nm, respectively. More in detail, the RMSF of the key residues Asn487, Lys417, Tyr449, and Thr500 of the S-RBD active site were less than 0.25 nm, indicating that they can interact with RN-4 strongly undergoing the simulation. The RMSF of the key residues of NRP1-BD binding with RN-4 in nm are Gly318 (0.065), Tyr297 (0.078), Trp301 (0.051), Asp320 (0.083), Ser346 (0.074), Thr349 (0.116), Tyr353 (0.057), and Ile415 (0.050), respectively. No much deviation was found in these amino acids, reflecting an intensive interaction with RN-4, which is consistent with the docking results above. Based on the results mentioned above, we can conclude that RN-4 matched well with the active sites of S-RBD and NRP1-BD, indicating the reasonable binding modes. DSSP analysis was further conducted to explore the alterations in the secondary structure of S-RBD and NRP1-BD. Figure 5(G, H) showed that no noticeable changes were found in structural elements undergoing the whole simulation, indicating the structural stability of S-RBD-RN-4 and NRP1-BD-RN-4 systems.

## Discussion

S-RBD and NRP1 are important in the SARS-CoV-2 entry. The inhibitors with dual inhibitory activities of S-RBD and NRP1 will be more effective to prevent SARS-CoV-2 infection. In this study, we successfully applied a multistep computer-aided protocol to discover five novel S-RBD/NRP1 dual-targeting peptides, all of which exhibited strong *in vitro* affinities in the low nanomolar range. Among them, RN-4 displayed the strongest binding affinity to both S-RBD and NRP1-BD, which was enhanced remarkably by about 135- and 304-fold compared with T-96 and EG00229. Therefore, it may be the most promising peptide targeting S-RBD and NRP1-BD. Further pseudotyped virus infection assay revealed that RN-4 showed obvious inhibitory effects against SARS-CoV-2 pseudovirus into 293 T cells (EC_50_ = 0.39 ± 0.09 μM) with negligible side effects on host cells. In addition, it did not show any detectable cellular toxicity to A549, AC16, and FHs-74 int cells, indicating its biological safety. Furthermore, the results of molecular docking suggested that Asn487, Lys417, Tyr449, and Thr500 are significant in the interactions between RN-4 and S-RBD to block the whole SARS-CoV-2 binding surface. Meanwhile, RN-4 could bind to NRP1 via serval hydrogen bonds interactions such as Gly318, Tyr297, Trp301, Asp320, Ser346, Thr349, Tyr353, and Ile415, thereby blocking the attachment between virus and host cells. Finally, molecular dynamics simulation indicated that RN-4 can stably bind to both S-RBD and NRP1-BD with no noticeable changes in the secondary structure of S-RBD and NRP1-BD. These results suggest that RN-4 is a dual S-RBD/NRP1-targeting, low-toxic, and high-efficacy anti-SARS-CoV-2 agent. However, given that linear peptides often possess low proteolytic stability, RN-4 may not be an ideal peptide for *in vivo* study. Further structural optimisation such as N-methylation of RN-4 backbone, cyclisation (stapled peptides, etc), and the replacement of an l-amino acid with a d-amino acid may be needed to improve the potency and pharmacokinetic properties of RN-4. In summary, we have successfully discovered of a dual S-RBD/NRP1-targeting peptide and it may become a starting point for further investigation of novel dual S-RBD/NRP1-targeting anti-SARS-CoV-2 drugs.

## Supplementary Material

Supplemental Material
