# Supplementary material for: Discovery of dual S-RBD/NRP1-targeting peptides: structure-based virtual screening, synthesis, biological evaluation, and molecular dynamics simulation studies
Source: J Enzyme Inhib Med Chem. 2023 May 17;38(1):2212327. doi: 10.1080/14756366.2023.2212327 (PMC10193894; doi:10.1080/14756366.2023.2212327)
Supplement: Supplemental Material [file IENZ_A_2212327_SM6434.pdf]

Supplementary Material

Discovery of dual S-RBD/NRP1-targeting peptides: structure-based virtual screening, synthesis, biological evaluation, and molecular dynamics simulation studies

Chunfang Hu[†,1], Ting Guo[†,2], Yunting Zou[†,3], Junyi Gao[1], Yi Gao[1], Miaomiao Niu[3], Yang Xia[*,1], Xiaozhou Shen[*,1], and Jindong Li[*,1]

[1]*Institute of Clinical Medicine, The Affiliated Taizhou People's Hospital of Nanjing Medical University, Taizhou, China*

[2]*Institute of Clinical Medicine, Taizhou People's Hospital Affiliated to Nanjing University of Traditional Chinese Medicine, Taizhou, China*

[3]*Department of Pharmaceutical Analysis, China Pharmaceutical University, Nanjing, China*

[†]*These authors contributed equally to this work.*

***Correspondence:**
Corresponding Author

camus_apollo@163.com (Yang Xia)

kevinsxz@163.com（Xiaozhou Shen）

warm4455@163.com (Jindong Li)

1. Table S1. Pharmacophore model validation using GH score method

Serial no.	Parameter	Pharmacophore model
1	Total molecules in database (*D*)	500
2	Total number of actives in database (*A*)	6
3	Total hits (*Ht)*	8
4	Active hits (*Ha*)	6
5	% Yield of actives[(*Ha*/*Ht*) × 100]	75%
6	% Ratio of actives [(*Ha*/*A*) × 100]	100%
7	Enrichment factor (*E*) [(*Ha* × *D*)/(*Ht* × *A*)]	63
8	False negatives [*A* - *Ha*]	0
9	False positives [*Ht* - *Ha*]	2
10	Goodness of hit score (*GH*)	0.81

2. Table S2. The docking scores of the screened peptides

Name	S-RBD	NRP1-BD
	Binding free energy[a] (kcal/mol)	Binding free energy (kcal/mol)
RN-1	-13.74	-13.15
RN -2	-13.68	-12.09
RN -3	-13.59	-11.65
RN -4	-14.15	-13.37
RN -5	-13.56	-12.53
T-96	−13.14	–
Peptide 5	−12.83	–

[a]Binding free energy between the ligands and the target (lower binding free energies suggest stronger binding affinities).

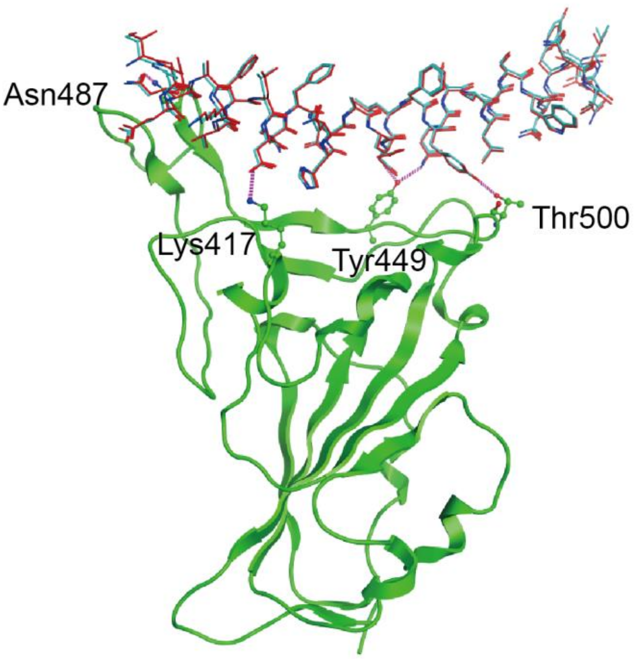

3. Figure S1. The docking conformation (cyan) and the original conformation (red) of the wild-type peptide (^{21}IEEQAKTFLDKFNHEAEDLFYQSSLASWNYNTNIT^{55}) derived from ACE2 protein in the active site of SARS-CoV-2 spike receptor-binding domain (S-RBD).

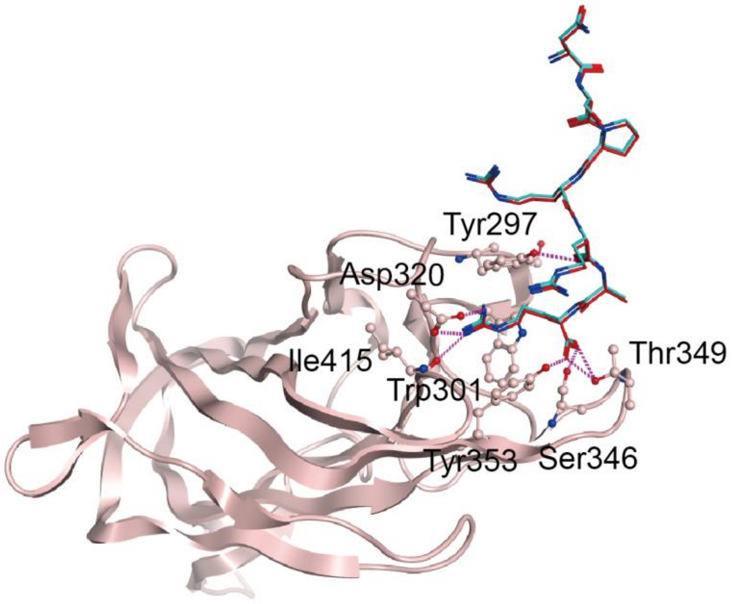

4. Figure S2. The docking conformation (cyan) and the original conformation (red) of the co-crystallized ligand CendR peptide (^{679}NSPRRAR^{685}) in the active site of NRP1.

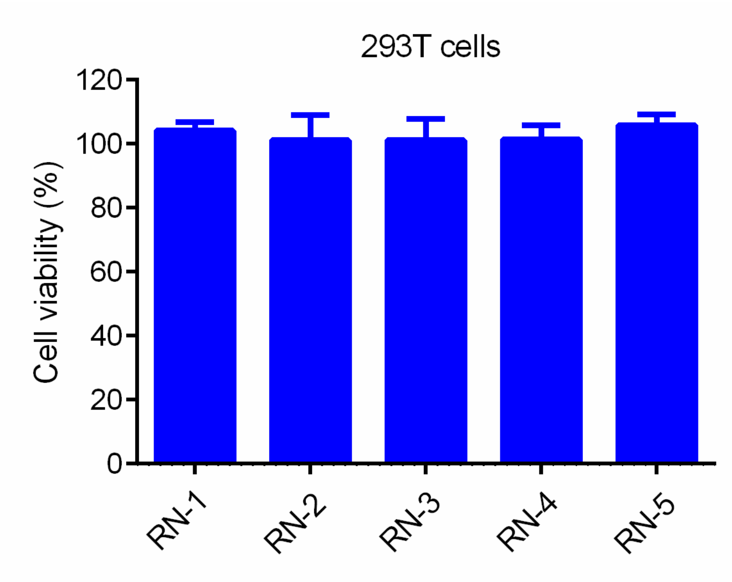

5. Figure S3. Effects of RNs 1-5 on the viability of 293T cells. Cells were treated with RNs 1-5 at a concentration of 30 μM for 48 h, and MTT assay was used to detect the cytotoxicity of peptides to cells. The results are represented as mean ± SD (n = 3).

6. HPLC chromatogram and MS spectrum of RNs 1-5

HPLC chromatogram of RN-1

HPLC REPORT

Sequence :Ac-QNKVYIDKYNHEAQDLFYQSALKAHRGNRRAR

Product Name :QR-32-1

Lot No :P220511-MX992905

Column :4.6×250mm, Sinochrom ODS-BP

Solvent A :0.1%Trifluoroacetic in 100% Acetonitrile

Solvent B :0.1%Trifluoroacetic in 100% Water

Gradient		A	B
	0.01min	20%	80%
	25min	45%	55%
	25.01min	100%	0%
	30min	Stop	

Flow rate :1.0ml/min

Wavelength :220nm

Volume :20ul

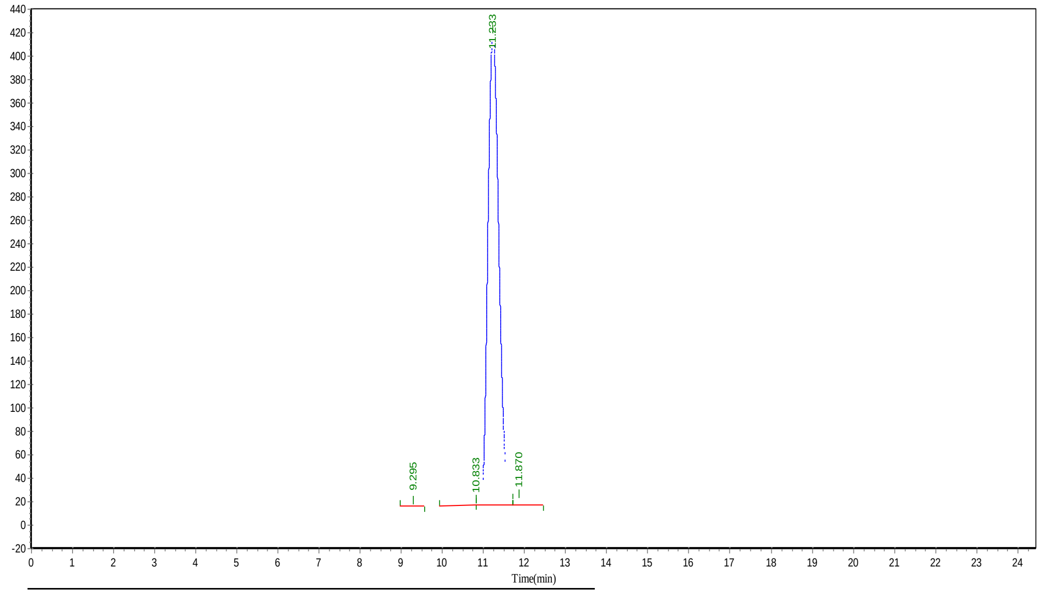

Rank	Time	Area	Conc.
1	9.295	7802.597	0.1027
2	10.833	24305.041	0.3200
3	11.233	7459474.500	98.2187
4	11.870	103176.938	1.3585
Total		7594759.076	100

MS spectrum of RN-1

MS Spectrum

Sequence : Ac-QNKVYIDKYNHEAQDLFYQSALKAHRGNRRAR

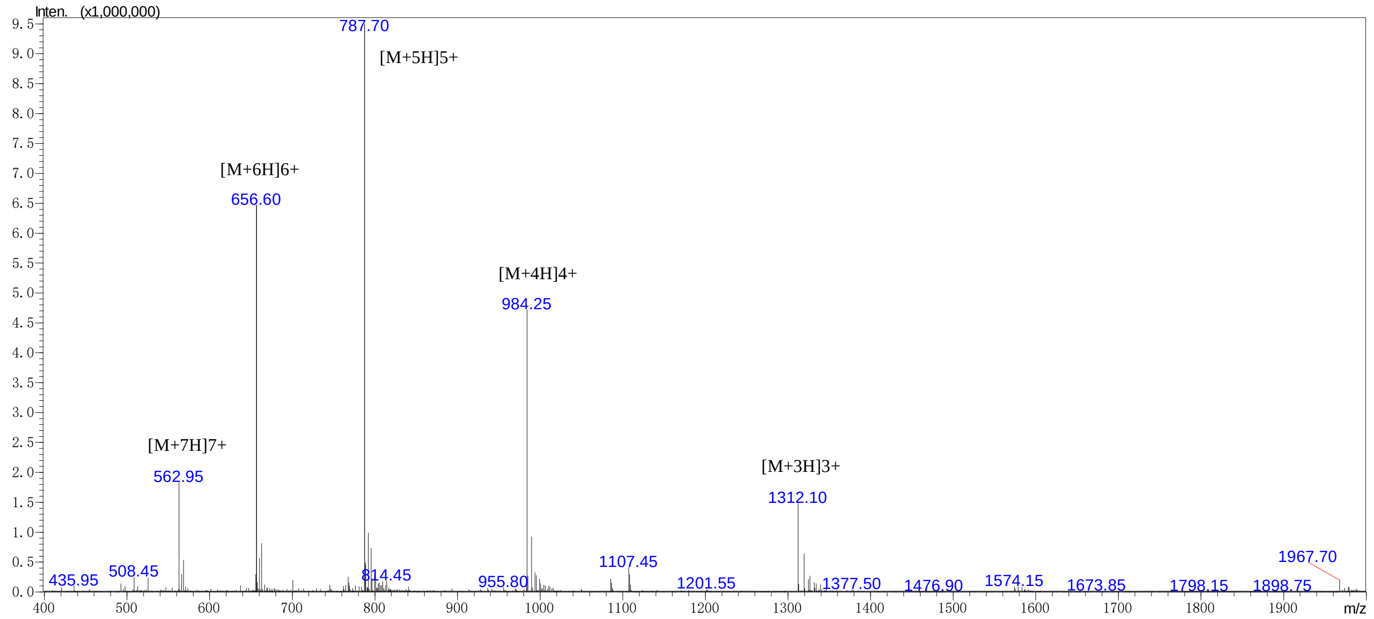

Date and Time:.2022/5/28
User : SHEN
Sample : QR-32-1
MW : 3933.31
Lot No. : P220511 -MX992905

Probe:	ESI	Probe bias:	+4.5kv
Nebulizer Gas Flow:	1.5L/min	Detector:	1.5kv
CDL:	-20.0v	T. Flow:	0.2ml/min
CDL Temp:	250℃	B. conc:	50%H2O/50%ACN
Block Temp:	200℃		

MS (ESI): theoretical mass: 3933.31, found $[M + 7H]^{7+}$ = 562.95, $[M + 6H]^{6+}$ = 656.60, $[M + 5H]^{5+}$ = 787.70, $[M + 4H]^{4+}$ = 984.25, $[M + 3H]^{3+}$ = 1312.10.

HPLC chromatogram of RN-2

HPLC REPORT

Sequence	:Ac-EKSTLKDRFNHEAQDLKYQDSLASYRGKRPPR
Product Name	:ER-32-2
Lot No	:P220511-MX992906
Column	:4.6×250mm,Sinochrom ODS-BP
Solvent A	:0.1%Trifluoroacetic in 100% Acetonitrile
Solvent B	:0.1%Trifluoroacetic in 100% Water

Gradient	:	A	B
	0.01min	18%	82%
	25min	43%	57%
	25.01min	100%	0%
	30min	Stop	

Flow rate	:1.0ml/min
Wavelength	:220nm
Volume	:20ul

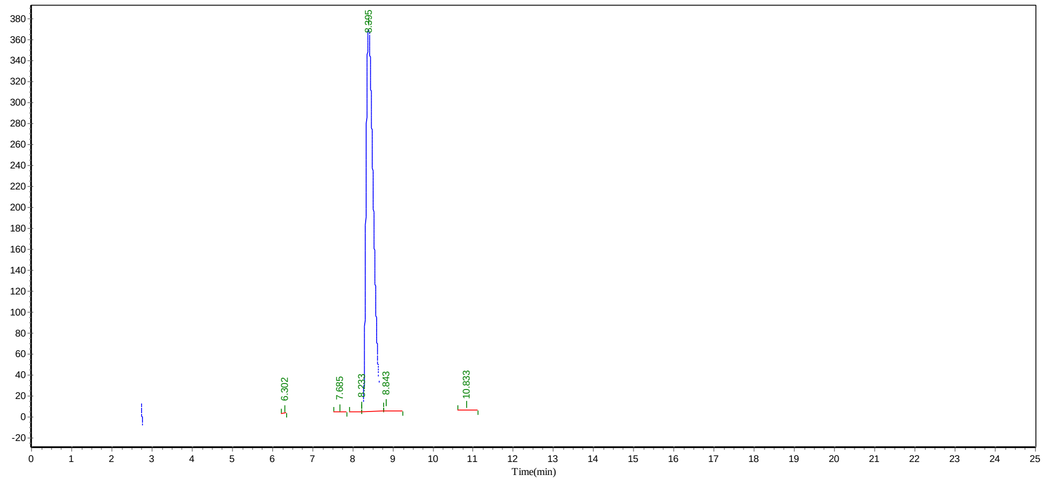

Rank	Time	Area	Conc.
1	6.302	1013.200	0.0216
2	7.685	5581.798	0.1192
3	8.233	12217.651	0.2609
4	8.395	4595801.500	98.1441
5	8.843	44991.352	0.9608
6	10.833	23103.809	0.4934
Total		4682709.310	100

MS spectrum of RN-2

MS Spectrum

Sequence : Ac-EKSTLKDRFNHEAQDLKYQDSLASYRGKRPPR

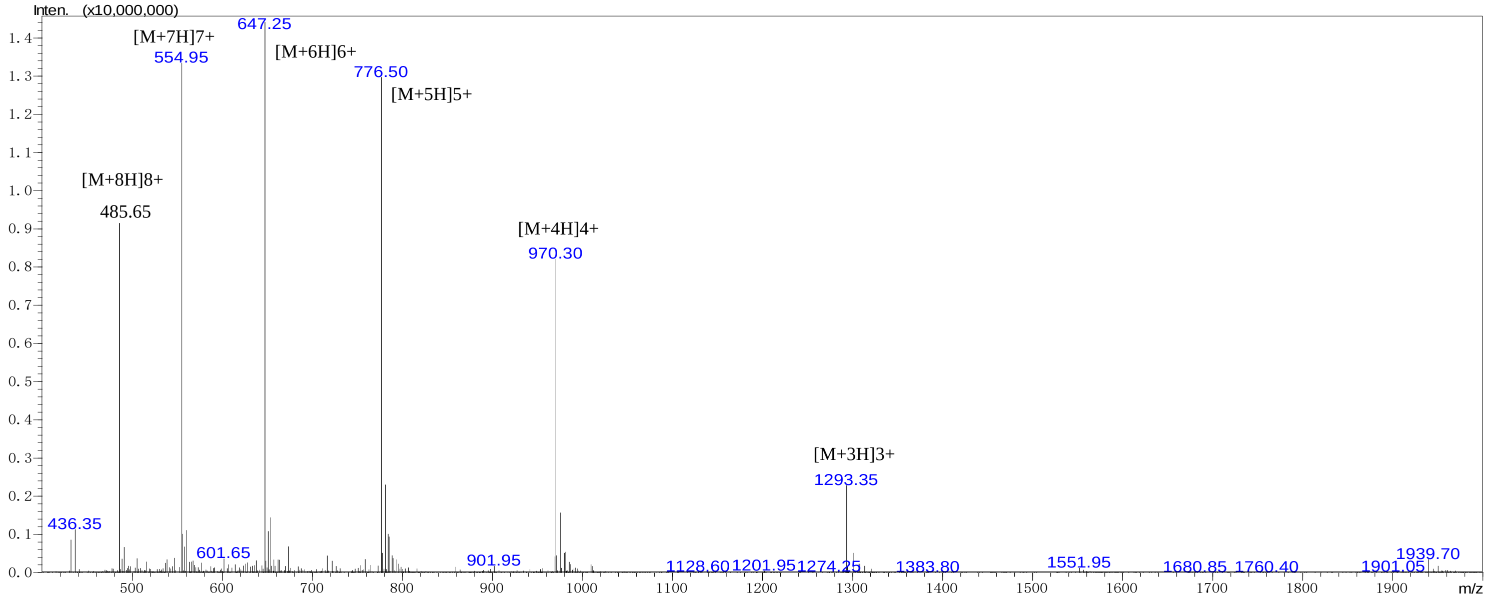

Date and Time:.2022/5/30
User : SHEN
Sample : ER-32-2
MW : 3877.24
Lot No. : P220511 -MX992906

Probe: ESI
Nebulizer Gas Flow: 1.5L/min
CDL: -20.0v
0.2ml/min
CDL Temp: 250℃
Block Temp: 200℃
Probe bias: +4.5kv
Detector: 1.5kv
T. Flow:
B. conc: 50%H2O/50%ACN

MS (ESI): theoretical mass: 3877.24, found [M + 8H] $^{8+}$ = 485.65, [M + 7H] $^{7+}$ = 554.95, [M + 6H] $^{6+}$ = 647.25, [M + 5H] $^{5+}$ = 776.50, [M + 4H] $^{4+}$ = 970.30, [M + 3H] $^{3+}$ = 1293.35.

HPLC chromatogram of RN-3

HPLC REPORT

Sequence	:Ac-QRNIYLDRHNHEAEDLKYQTALASFRPAKPAR
Product Name	:QR-32
Lot No	:P220511-MX992907
Column	:4.6×250mm, Sinochrom ODS-BP
Solvent A	:0.1%Trifluoroacetic in 100% Acetonitrile
Solvent B	:0.1%Trifluoroacetic in 100% Water

Gradient	:	A	B
	0.01min	19%	81%
	25min	44%	56%
	25.01min	100%	0%
	30min	Stop	

Flow rate	:1.0ml/min
Wavelength	:220nm
Volume	:20ul

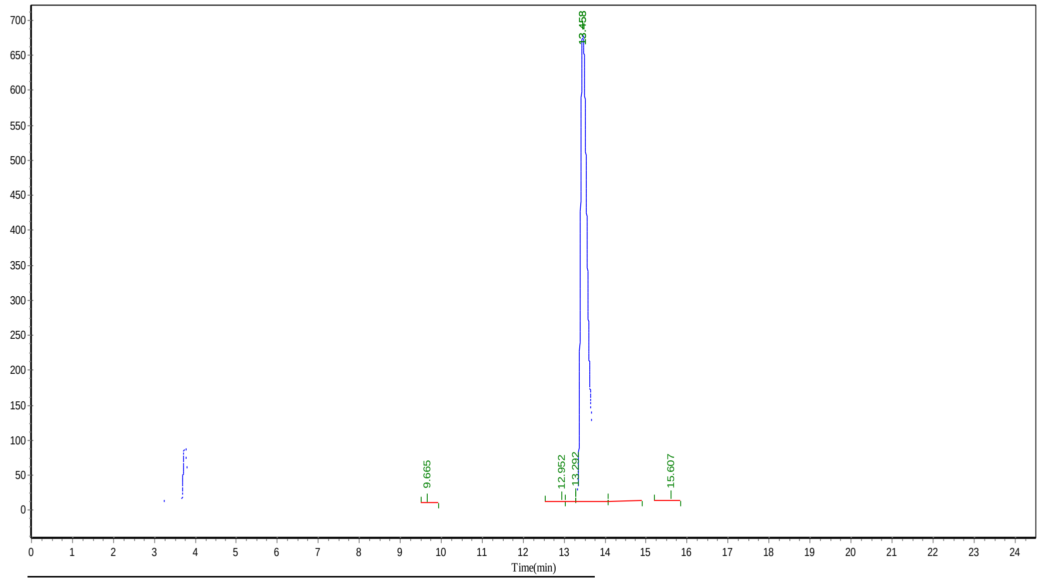

Rank	Time	Area	Conc.
1	9.665	8670.116	0.1035
2	12.952	32008.355	0.3821
3	13.292	46217.215	0.5517
4	13.458	8226364.000	98.1968
5	13.458	42755.543	0.5104
6	15.607	21413.910	0.2556
Total		8377429.139	100

MS spectrum of RN-3

MS Spectrum

Sequence : Ac-QRNIYLDRHNHEAEDLKYQTALASFRPAKPAR

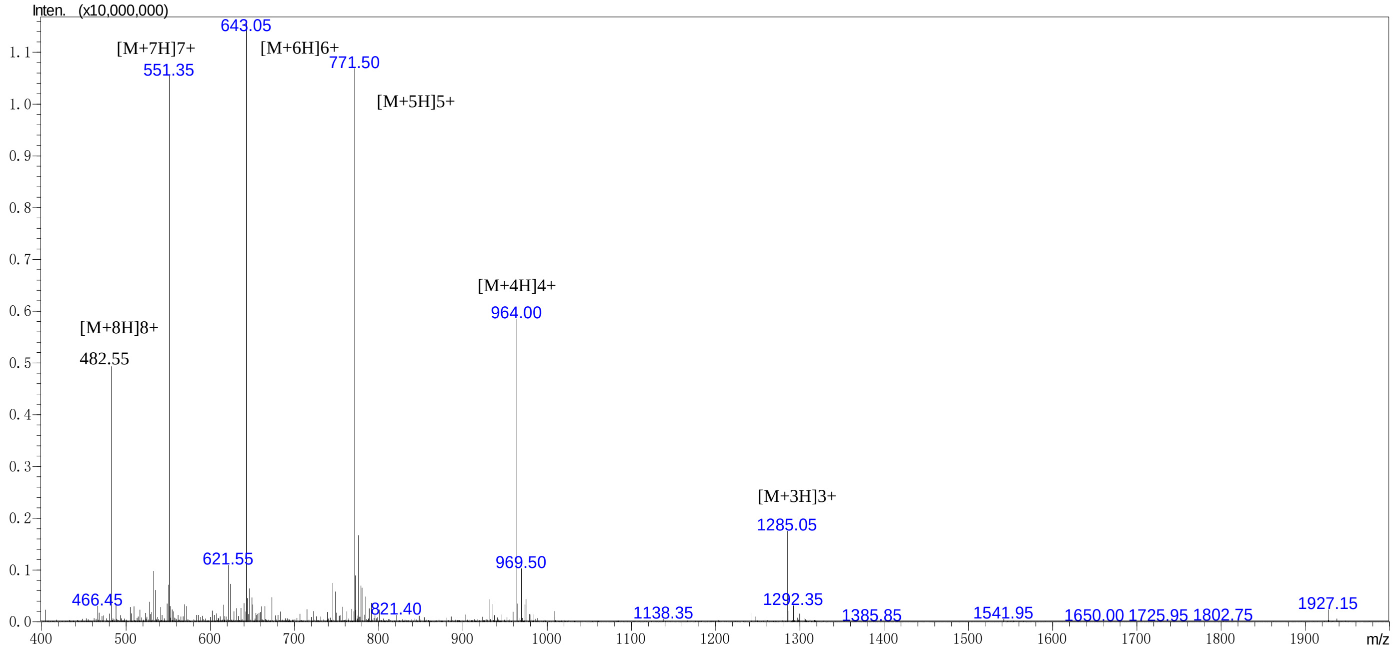

Date and Time:.2022/5/28
User : SHEN
Sample : QR-32
MW : 3852.23
Lot No. : P220511 -MX992907

Probe:	ESI	Probe bias:	+4.5kv
Nebulizer Gas Flow:	1.5L/min	Detector:	1.5kv
CDL:	-20.0v	T. Flow:	0.2ml/min
CDL Temp:	250℃	B. conc:	50%H2O/50%ACN
Block Temp:	200℃		

MS (ESI): theoretical mass: 3852.23, found $[M + 8H]^{8+}$ = 482.55, $[M + 7H]^{7+}$ = 551.35, $[M + 6H]^{6+}$ = 643.05, $[M + 5H]^{5+}$ = 771.50, $[M + 4H]^{4+}$ = 964.00, $[M + 3H]^{3+}$ = 1285.05.

HPLC chromatogram of RN-4

HPLC REPORT

Sequence	:AC-EARTFLDKYNHEAEDLYYQSSLASWGSPRRAR		
Product Name	:ER-32-1		
Lot No	:P220511-MX992908		
Column	:4.6×250mm, Sinochrom ODS-BP		
Solvent A	:0.1%Trifluoroacetic in 100% Acetonitrile		
Solvent B	:0.1%Trifluoroacetic in 100% Water		
Gradient	:	A	B
	0.01min	25%	75%
	25min	50%	50%
	25.01min	100%	0%
	30min	Stop	
Flow rate	:1.0ml/min		
Wavelength	:220nm		
Volume	:20ul		

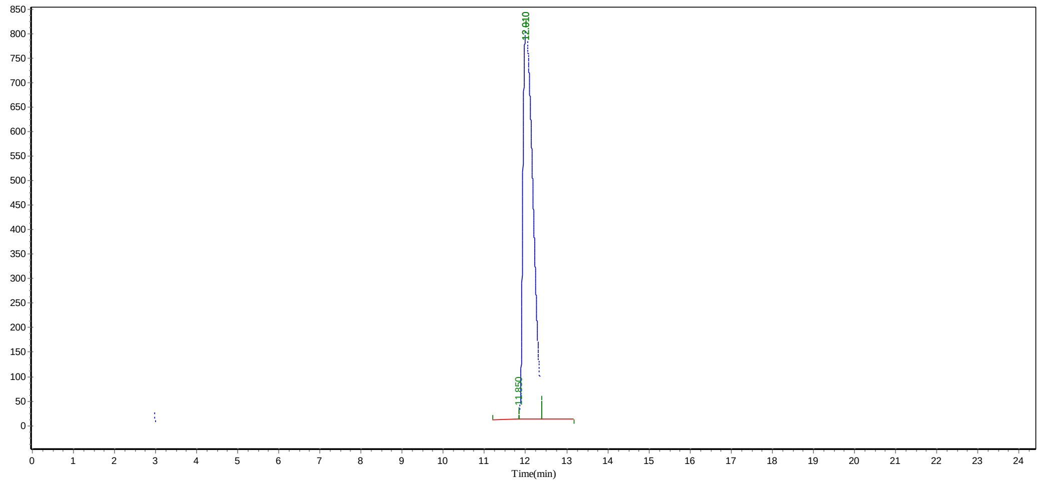

Rank	Time	Area	Conc.
1	11.850	42449.051	0.3090
2	12.010	13491695.000	98.2045
3	12.010	204222.531	1.4865
Total		13738366.582	100

MS spectrum of RN-4

MS Spectrum

Sequence : Ac-EARTFLDKYNHEAEDLYYQSSLASWGSPRRAR

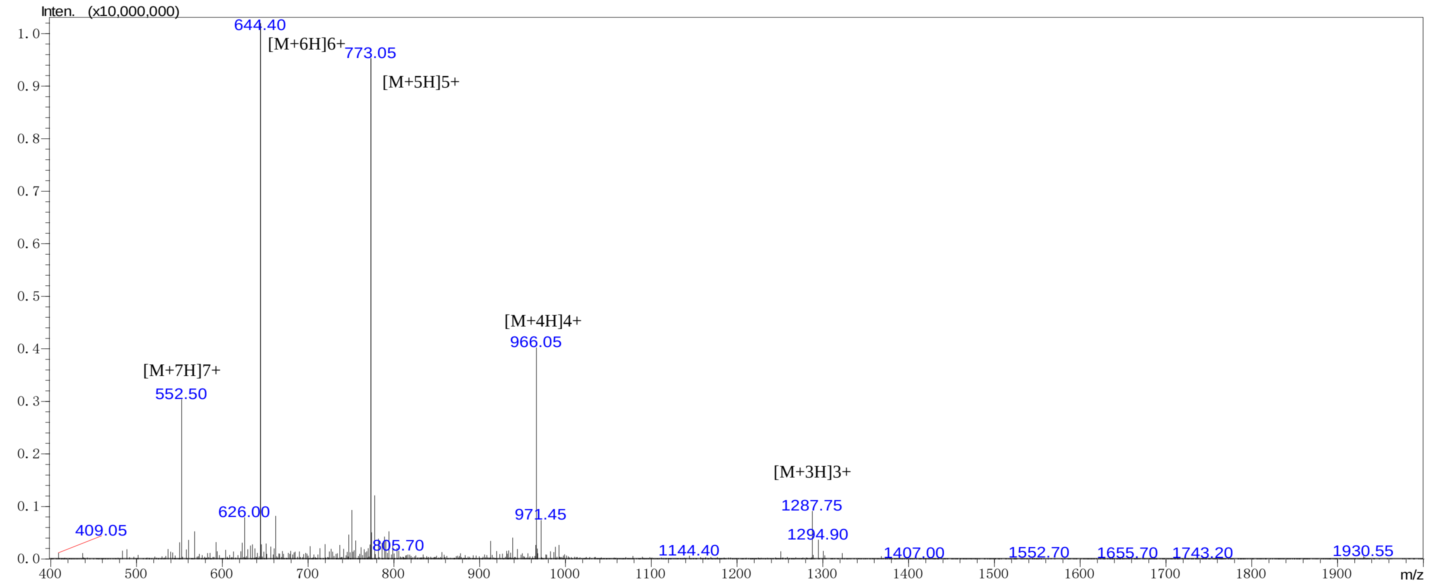

Date and Time:.2022/5/30		Probe:	ESI	Probe bias:	+4.5kv
User	: SHEN	Nebulizer Gas Flow:	1.5L/min	Detector:	1.5kv
Sample	: ER-32-1	CDL:	-20.0v	T. Flow:	
MW	: 3860.12		0.2ml/min		
Lot No.	: P220511 -MX992908	CDL Temp:	250℃	B. conc:	50%H2O/50%ACN
		Block Temp:	200℃		

MS (ESI): theoretical mass: 3860.12, found [M + 7H] $^{7+}$ = 552.50, [M + 6H] $^{6+}$ = 644.40, [M + 5H] $^{5+}$ = 773.05, [M + 4H] $^{4+}$ = 966.05, [M + 3H] $^{3+}$ = 1287.75.

HPLC chromatogram of RN-5

HPLC REPORT

Sequence	:Ac-ETQVLLDKLNHEAEDLKYQSALASHRVRRPPR
Product Name	:ER-32
Lot No	:P220511-MX992909
Column	:4.6×250mm, Sinochrom ODS-BP
Solvent A	:0.1%Trifluoroacetic in 100% Acetonitrile
Solvent B	:0.1%Trifluoroacetic in 100% Water

Gradient	:	A	B
	0.01min	25%	75%
	25min	50%	50%
	25.01min	100%	0%
	30min	Stop	

Flow rate	:1.0ml/min
Wavelength	:220nm
Volume	:20ul

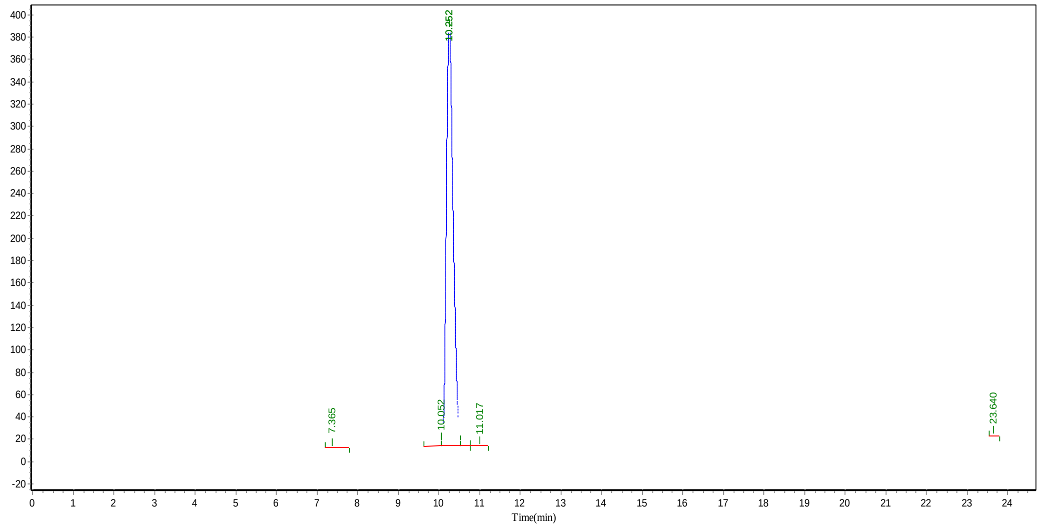

Rank	Time	Area	Conc.
1	7.365	9676.303	0.2181
2	10.052	24569.252	0.5538
3	10.252	4368005.500	98.4491
4	10.252	14521.323	0.3273
5	11.017	10635.814	0.2397
6	23.640	9406.909	0.2120
Total		4436815.101	100

MS spectrum of RN-5

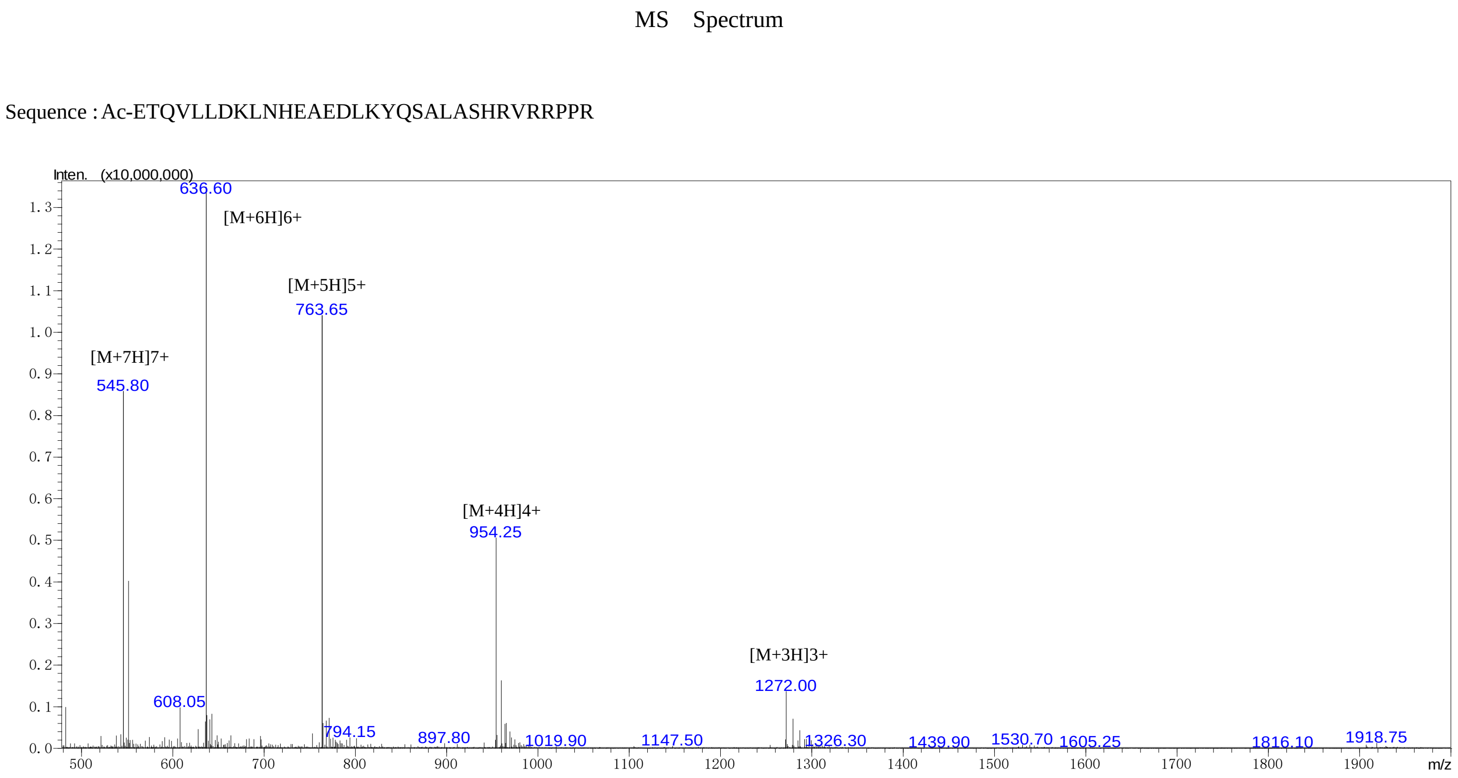

Date and Time:.2022/5/30		Probe:	ESI	Probe bias:	+4.5kv
User	: SHEN	Nebulizer Gas Flow:	1.5L/min	Detector:	1.5kv
Sample	: ER-32	CDL:	-20.0v	T. Flow:	
			0.2ml/min		
MW	: 3813.24	CDL Temp:	250℃	B. conc:	50%H2O/50%ACN
Lot No.	: P220511 -MX992909	Block Temp:	200℃		

MS (ESI): theoretical mass: 3813.24, found $[M + 7H]^{7+}$ = 545.80, $[M + 6H]^{6+}$ = 636.60, $[M + 5H]^{5+}$ = 763.65, $[M + 4H]^{4+}$ = 954.25, $[M + 3H]^{3+}$ = 1272.00.
